# Bidirectional Cerebellar Control of Suprasecond Timing in Rats

**DOI:** 10.1523/ENEURO.0198-25.2025

**Published:** 2026-02-03

**Authors:** Ellen Boven, Jasmine Pickford, Richard Apps, Nadia L. Cerminara

**Affiliations:** ^1^Department of Neuroscience, Erasmus MC, Rotterdam 3015 AA, The Netherlands; ^2^School of Physiology, Pharmacology and Neuroscience, Faculty of Life Sciences, University of Bristol, Bristol BS8 1TH, United Kingdom

**Keywords:** cerebellum, prediction, subsecond, suprasecond, timing

## Abstract

The cerebellum is well established in subsecond motor timing, but its role in suprasecond interval timing remains unclear. Here, we investigated how cerebellar output influences time estimation over longer timescales. Male rats performed a nose-poke interval timing task in which reward availability could be predicted either from a fixed 2.5 s auditory cue (cued trials) or had to be estimated internally during uncued 3.5 s trials that demanded self-timing. Chemogenetic inhibition of the lateral cerebellar nucleus (LCN) produced bidirectional effects: delayed action initiation in predictable trials and premature (∼100–160 ms) responses when self-timing was required. Despite a slowing of movement, overall task success rates remained unchanged. Because motor slowing is likely to lead to later, not earlier, action initiation, these results implicate the LCN in computing internal time estimates. These findings demonstrate that the cerebellum integrates motor and cognitive processes for suprasecond timing, with differential effects on externally guided and self-generated timing.

## Significance Statement

The cerebellum, a brain region best known for fine-tuning movements with subsecond accuracy, may also be involved in judging longer time intervals. Rats were trained on an interval timing task where suprasecond auditory tones signaled either a predictable external cue or an unpredictable cue that required estimating time that was dependent on self-timing. By reversibly inhibiting lateral cerebellar output, we show that rats misjudge time over seconds, either over or underestimating time depending on whether they rely on an external cue or on self-timing. Locomotor slowing was modest and success rates were preserved, dissociating temporal estimation from execution deficits. The findings are therefore consistent with the cerebellum contributing to both subsecond motor and suprasecond cognitive self-timing processes.

## Introduction

The ability to produce sequential movements underlies everyday behavior, with timing serving as a vital component in motor control (Avanzino et al., 2016). External stimuli provide temporal cues to enable coordination with moving objects in dynamic environments, while an internal perception of time intervals is required when participants estimate time duration and execute a task or compare the duration to a reference time interval (Grondin, 2010). Self-timing—the ability to initiate movements based on an internally generated temporal framework rather than external sensory cues—is a key aspect of this process.

Timing tasks can also be divided by their temporal scale (Buhusi and Meck, 2005; Issa et al., 2020; Zhou and Buonomano, 2022). Subsecond timing tasks, i.e., those that operate within millisecond intervals, include rapid motor functions like locomotion, reflexes, eye movement, and object manipulation—functions that are dependent on an intact cerebellum (Krupa and Thompson, 1997; Garcia and Mauk, 1998; Mojtahedian et al., 2007). Conversely, suprasecond timing, which operates over intervals ranging from seconds to minutes, is associated with goal-directed behaviors requiring higher cognitive processes such as foraging and decision-making (Gupta, 2014; Pezzulo et al., 2014; Friedman and Robbins, 2022; Szelag et al., 2022). While much is known about cerebellar contributions to subsecond timing (for reviews see Breska and Ivry, 2016; Tanaka et al., 2021; Ivry et al., 2022; Boven and Cerminara, 2023), its contributions to suprasecond timing remain poorly understood.

Human studies, including cerebellar patients and functional brain imaging, have demonstrated the possible role of the cerebellum in suprasecond timing (Nichelli et al., 1996; Malapani et al., 1998; Mangels et al., 1998; Tracy et al., 2000; O’Reilly et al., 2008; Gooch et al., 2010). Suprasecond timing is often studied through behavioral paradigms of interval timing, which require individuals to monitor time over several seconds to minutes to guide goal-directed actions and decision-making. These behaviors involve cognitive components, where individuals estimate and act on time intervals that are longer than those typically studied in cerebellar research yet have been instrumental in demonstrating the potential role of the cerebellum in suprasecond timing. For example, Gooch et al. (2010) observed that cerebellar patients with damage to the lateral regions of the cerebellum exhibited both overestimation and underestimation of suprasecond intervals during temporal estimation and production tasks. However, given the inherent methodological constraints of human studies, it remains unclear how the cerebellum contributes to these behaviors.

Interval timing tasks in animals can provide insight into how the cerebellum facilitates temporal estimation while controlling for motor performance (Parker, 2016; Ohmae et al., 2017; Kunimatsu et al., 2018; Heskje et al., 2020; Heslin et al., 2022). Ohmae et al. (2017) and Kunimatsu et al. (2018) examined the role of the cerebellar dentate nucleus in self-timing by training monkeys to initiate saccades after delay intervals ranging from 0.4 to 2.4 s. Their findings demonstrated that preparatory neural activity in the dentate nucleus preceding self-timed saccades was temporally consistent across delay intervals, irrespective of duration, suggesting that the cerebellar dentate nucleus may contribute not only to subsecond timing but also to the execution and fine-tuning of movements following suprasecond delays. This contrasts with findings from Heslin et al. (2022), who found no evidence for cerebellar involvement in suprasecond interval timing tasks in rodents. The discrepancy between these studies highlights ongoing uncertainty regarding the cerebellum's role in self-timing beyond the subsecond range. Therefore, the aim of the present study was to investigate cerebellar contributions to suprasecond self-timing by utilizing an interval timing task in rats while controlling for motor strategies. In this task, rats were required to estimate time based on sound duration, terminating a nose poke at the appropriate moment to receive a reward. Previous research has shown that this task recruits the prefrontal cortex but not the motor cortex (Xu et al., 2014), suggesting recruitment of higher cognitive processes in the task. Using a suprasecond time estimation task and inhibitory chemogenetics in the lateral cerebellar nucleus (LCN; rodent homolog of dentate), we show that rats underestimate time, suggesting cerebellar contributions to self-timing at suprasecond intervals. These findings therefore support the role of the cerebellum in integrating motor and cognitive functions to enable adaptive behavior over extended timescales.

## Materials and Methods

### Experimental animals

All animal procedures were performed in accordance with the UK Animals (Scientific Procedures) Act 1986 and were approved by the University of Bristol Animal Welfare and Ethical Review Body (PPL number PA26B438F). Experiments were conducted on 20 male Lister hooded rats (HsdOla:LH, 330–480 g at the time of surgery, Envigo). Animals were housed in pairs under a 12:12 h reverse light/dark cycle (light phase 20:15–08:15; target conditions, 20°C and 45–65% humidity). Experiments were therefore performed during the dark phase when they are naturally most active. Food and water were available *ad libitum* prior to and during recovery from surgical procedures. Water was available *ad libitum* throughout the experiment. After recovery from surgical procedures (see below), animals were each fed ∼16 g of standard laboratory chow per day, in addition to food rewards obtained during behavioral tasks. For tasks involving food reward, 45 mg grain-based sweetened reward pellets (TestDiet LabTab AIN-76, 5TUL, catalog #1811155) were used. The weights of the animals were monitored 5 d a week to ensure they did not drop below 90% of the normal growth curve. Handling occurred daily 1 week prior to surgery and during the recovery phase. Animals were habituated to the experimenter and experimental room for 5 consecutive days prior to behavioral training. During the experiment, animals were handled every weekday.

### Viral vectors

Viral vectors were injected into the LCN in order to study the role of cerebellar projections during an interval timing estimation task. Two AAV vectors were used: 10 animals received bilateral injections of designer receptors exclusively activated by designer drugs (DREADD) virus [AAV5-hSyn-hM4D(Gi)-mCherry, Addgene, plasmid #50465; titer 1.2 × 10^12 ^gc.ml], whereas 10 animals received bilateral injections of a control virus (AAV5-hSyn-EGFP, Addgene, plasmid #50465; titer 1.2 × 10^12 ^gc.ml). Animals were randomly assigned to the control or the treatment group (hM4Di group). The experimenter was blinded to the identity of the virus used for transfection in each animal until after the behavioral analysis was complete.

### Surgical procedure

All surgical procedures were performed under aseptic conditions. General anesthesia was induced by initially administering gaseous isoﬂurane, followed by an intraperitoneal injection of ketamine (50 mg.kg^−1^; Vetalar) and medetomidine (0.3 mg.kg^−1^; Domitor). The depth of anesthesia was regularly monitored throughout surgery by testing the hindpaw withdrawal reﬂex, and additional doses of ketamine/medetomidine were given as necessary to maintain surgical anesthesia. Each animal was placed in a stereotaxic frame with atraumatic ear bars. Throughout the procedure, body temperature was maintained at ∼37°C with the aid of a thermostatically controlled heated blanket. Eye ointment (Lacri-Lube) was placed on the eyes to prevent corneal injury due to drying. The incision site was treated with local anesthetic cream (lidocaine). A midline scalp incision was made to access the skull. Bregma and lambda were measured to ensure the skull was level in the dorsoventral plane. Coordinates relative to bregma were measured to allow precise positioning of burr holes for viral injections. The LCN injections were performed bilaterally at AP −11.2 mm and ML ±3.4 mm relative to the bregma and DV −4.0 mm from the surface of the cerebellum. Virus was delivered using a pulled glass micropipette connected to a 25 µl syringe (Hamilton) via tubing ﬁlled with mineral oil and was then backﬁlled with 1 µl of the viral vector using a syringe driver (AL-1000, World Precision Instruments). A volume of 0.5 nl was injected per hemisphere at 200 nl/min and the pipette then left in place for ∼10 min following viral delivery, to minimize leakage of the virus back up the pipette track. At the end of each surgery, rats were given the medetomidine antidote atipamezole (Antisedan, 0.1 mg, i.p.), analgesic (Metacam, 1 mg/kg, s.c.), and saline (10 ml/kg, s.c.). Rats were singly housed for 7 d following surgery and then returned to their original pairings. A minimum period of 6 weeks was allowed for expression of the viral vector before any experimental manipulations. During this period, animals underwent behavioral training on the interval time estimation task.

### Interval timing task

The interval time estimation task was performed in a standard light-resistant and sound-proof operant box (Med Associates) which were controlled by the K-Limbic software (Med Associates). The operant chamber consisted of two nose ports, and each port contained a light at the top and a nose-poke receptacle with an infrared (IR) photobeam at the bottom. The light was used as a reinforcer during the behavioral paradigm. The photobeam served as a head entry detector for nose pokes in each port and was created by a single IR light source and receiver. When the beam was uninterrupted, the IR receiver maintained a high output signal; when the rat's head entered the port, the IR beam would be broken; and the receiver would set the output signal to low. The left port served as the “hold port” in which the rats maintained their head position, while the other port served as the “reward port” for reward collection. Relative positions of the hold and reward port were ﬁxed across the whole experiment. A rubber tube connected the pellet receptacle of the reward port to the pellet dispenser. An audio generator produced tones that were delivered via a speaker placed at the top of the chamber, in between the two ports. The signals from the operant box were recorded through input/output cards and interfaced with a computer. Animal behavior was monitored via a camera (Microsoft LifeCam HD-3000) which was attached to the ceiling of the operant chamber above the hold port. In the interval timing task, food-restricted rats learnt to estimate a 2.5 s sound duration through positive reinforcement. Rats indicated the estimated time by exiting from the hold port; if this action was around the target duration of 2.5 s following tone onset, rats could nose poke into the reward port which would trigger delivery of a food pellet. During the auditory cue, rats maintained head placement within the hold port, which meant that gross movements such as locomotion and grooming were absent during the period when timing was evaluated.

The behavioral protocol used for this experiment is based on the interval timing task described by Xu et al. (2014). Rats were habituated to the operant box in two successive sessions of 20 min per session. Animals underwent training in stages as follows:

1. Tone training: The ﬁrst stage of the task required the rats to learn to associate a nose poke into the hold port with a 2.5 s auditory cue (white noise, 75 dB) and that a food pellet was delivered in the reward port on termination of the auditory cue. Criterion for the ﬁrst stage of training was 100 rewarded trials in 30 min on 2 consecutive days.
2. Action suppression training: Rats then progressed to the second stage where they learnt to actively hold a nose poke for 2.5 s during the auditory cue. The duration of the cue was gradually increased from 0.5 s to the target duration of 2.5 s according to individual performance. Successful holds resulted in a food pellet reward, whereas failure to sustain a nose poke led to a time-out, which consisted of the lights in both the hold and reward ports illuminated for 16 s. Criterion for this second stage was to sustain the nose poke for the required duration for at least 50% of the trials across two sessions, with each session consisting of a minimum of 100 trials. All rats learned the initial training Stages 1 and 2 within 2 weeks.
3. Interval timing training with predictable time cue (Fig. 2*A*): The next stage of training introduced the reward window and random delay. For each trial, the sound duration was ﬁxed to 2.5 s (Fig. 2*A*, black bar) and served as the cue for the rat to exit from the hold port at sound offset. As the sound offset conveys predictive information for reward availability, this is referred to as a time cue (Freestone et al., 2013). A random delay (drawn from a uniform distribution within 0.5–1.5 s) was introduced between the self-initiated nose poke and sound onset. This required the animal to pay attention to the cue, as the time for reward availability became contingent on the presentation of the cue and not on the nose poke. Exiting the hold port during the random delay was referred to as a “too early” trial and resulted in a time-out (Fig. 2*A*, gray bar). The reward window began 2.25 s after the start of the auditory cue and lasted 1 s after the end of the cue to accurately shape behavior, i.e., the reward window was from 2.25–3.5 s after the start of the tone (Fig. 2*A*, green bar). Nose-poke release during this time triggered a reward pellet and resulted in a “correct trial.” The animals had a duration of 1.5 s to collect their reward following release from the hold port (Fig. 2*A*). After reward collection, a new trial could be initiated after an intertrial interval of 6 s. Nose-poke release that occurred after stimulus onset but before the reward window resulted in an “incorrect trial” and a time-out period (Fig. 2*A*, red bar). Nose-poke releases that occurred beyond the reward window were not rewarded and considered “too late” trials (Fig. 2*A*, blue bar). Each session lasted 60 min in which the rats performed 150–200 trials.
4. Interval timing with unpredictable cue (Fig. 2*C*): After animals were trained and tested on the predictable time cue, animals moved onto the ﬁnal stage where trials with the predictable time cue of 2.5 s occurred randomly in 50% of the trials, and the other 50% of trials consisted of the same sound played for a duration of 3.5 s (Fig. 2*C*, black bars), but the reward window remained the same as in predictable time cue sessions (2.25–3.5 s; Fig. 2*C*, green bar). This stage of the training therefore resulted in unpredictable trials, because the exit time from the hold port was not reliably cued by the offset of the sound. Rats’ ability to estimate time independently of offset of an external sensory cue was therefore assessed in these sessions. Each session lasted 60 min, during which the rats completed 150–200 trials.

### Open ﬁeld

Open-ﬁeld behavioral testing was performed to determine if chemogenetic inhibition of cerebellar output had any general effect on motor performance. Open-ﬁeld exploration was assessed 30 min following clozapine *N*-oxide (CNO) injection. Open-ﬁeld testing was separate from behavioral testing on the interval timing task. Rats were placed at the perimeter of a cylindrical arena (90 cm diameter, 51 cm height) which was placed on a black matte plastic base on the ﬂoor. Rats were allowed to freely explore the arena for 10 min. The behavioral testing occurred in white light (∼140 lux). Behavior was monitored by an overhead webcam at 30 frames per second (fps). The arena was cleaned with 70% ethanol between each animal.

### CNO administration

CNO administration prior to behavioral was testing performed ﬁrst on the interval time estimation task with predictable time cue and then on the interval time estimation task with unpredictable time cue. All animals were injected intraperitoneally using a single handed modiﬁed restrained method to minimize stress and improve welfare (Stuart and Robinson, 2015). CNO (Tocris Bioscience) was administered at a dose of 2.5 mg/kg and dissolved in 5% DMSO and then diluted in 0.9% NaCl to a ﬁnal concentration of 2.5 mg/ml. An equivalent vehicle consisted of 0.9% saline in 5% DMSO. Behavioral testing began 30 min following the injection. Typically, testing was carried out over 5 d with Days 1, 3, and 5 consisting of baseline sessions and Days 2 and 4 consisting of CNO or vehicle administration. The experimenter was blinded to which treatment was administered until after the analysis had taken place.

### Histology and immunostaining

Upon completion of the experiments, animals were anesthetized with a lethal dose of Euthatal (200 mg/kg) and transcardically perfused with 0.9% saline followed by 4% paraformaldehyde. Each brain was dissected and postﬁxed in 4% paraformaldehyde. After several days, brains were transferred to 30% sucrose in 0.1 M phosphate buffer (PB) and stored until sectioning. Prior to being cut, the cerebellum was removed and embedded in gelatin. A freezing microtome (SM2000R, Leica) using Cryomatrix embedding medium (Thermo Fisher Scientiﬁc) was used to cut the cerebellum into 40 µm sagittal sections. Sections were collected in 0.01 M PB for preservation and prepared for immunohistochemistry to visualize viral expression of the control or hM4D(Gi) virus. In brief, sections were washed three times for 10 min in 0.01% PBS and placed in 50% ethanol for 30 min. After a further 3 × 10 min washes, sections were incubated overnight at room temperature in a chicken anti-eGFP (Abcam ab13970 at 1:2,000) or rabbit anti-mCherry (Biovision 5993-100 at 1:2,000) antibody containing 5% normal horse serum. The following day, sections were washed three times, for 10 min per wash, and incubated for 2 h with secondary antibody (Jackson ImmunoResearch Laboratories goat anti-chicken Alexa Fluor 488 or donkey anti-rabbit Alexa Fluor 594 both 1:1,000 in PBS-T). Sections were washed for 5 min in PBS before mounting on glass slides using 1% gelatin and 0.1% chromium potassium sulfate solution. Fluoromount with DAPI, a stain for all cell nuclei, was applied to the slides before they were coverslipped to prepare for imaging.

### Microscopy

**Figure 1.**
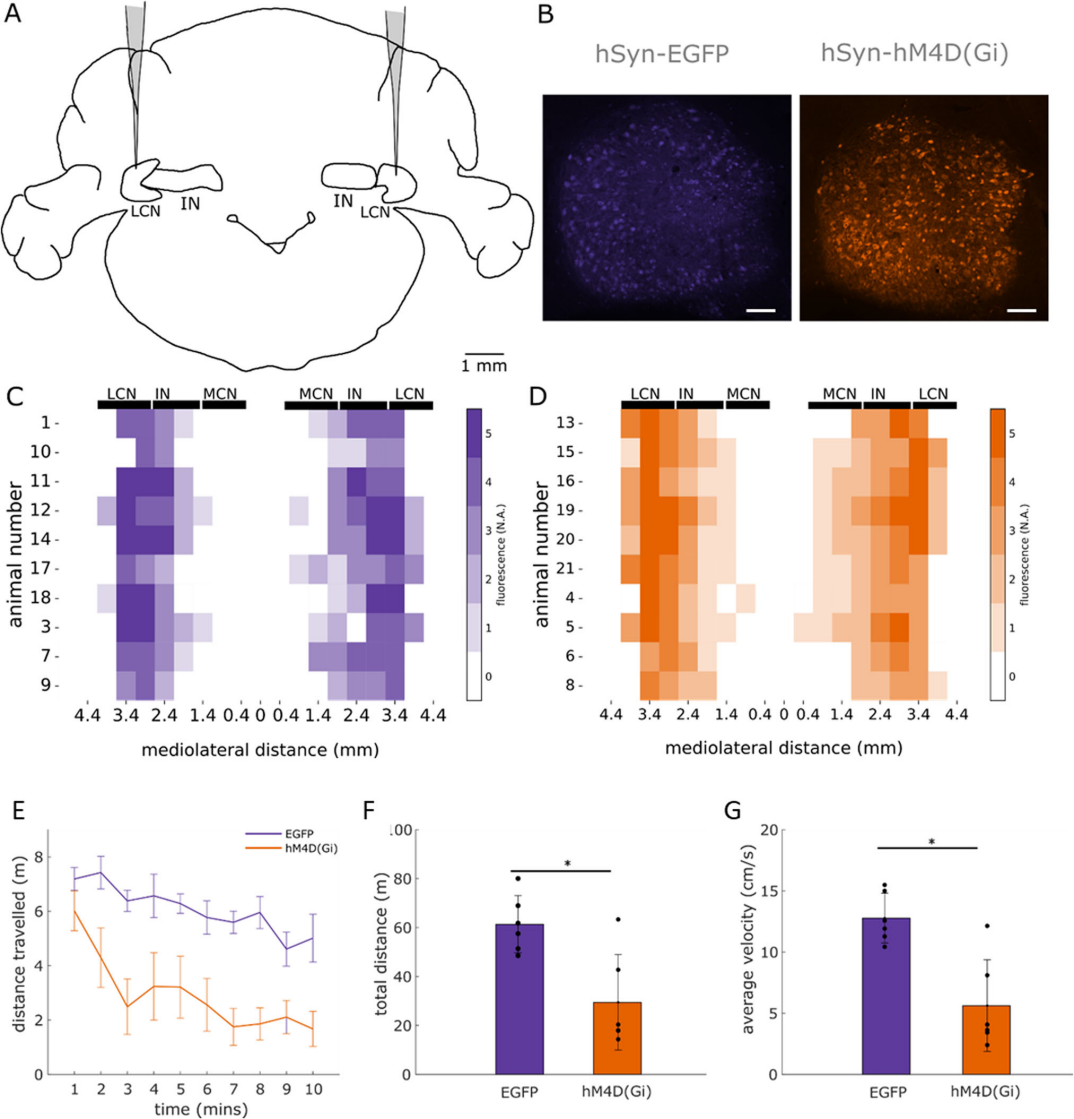
Viral expression in LCN and locomotor control. ***A***, Schematic of viral-mediated delivery of AAV5-hSyn-EGFP or AAV5-hSyn-hM4D(Gi) bilaterally targeting the LCN. ***B***, Sagittal sections of LCN showing representative examples of expression of AAV5-hSyn-EGFP and AAV5-hSyn-hM4D(Gi). Scale bars, 100 μm. ***C***, Heatmaps showing the extent of EGFP (*n* = 10) expression across the mediolateral axis of the cerebellar nuclei (LCN, lateral cerebellar nucleus; IN, interpositus nucleus; MCN, medial cerebellar nucleus). Colors of heatmap indicate the level of expression with no expression at 0 and maximal expression at 5. ***D***, Same as ***C*** but for hM4D(Gi) (*n* = 10). ***E***, Average distance traveled during the 10 min of open-field exploration for EGFP (purple, *n* = 6) and hM4D(Gi) (orange, *n* = 6) rats per minute. ***F***, Average total distance traveled during the 10 min of open-field exploration for EGFP (*n* = 5) and hM4D(Gi) (*n* = 6) groups. Individual data points show total distance traveled per animal. Plots in ***E*** and ***F*** represent mean ± SD. Unpaired *t* test *, *p* < 0.01. ***G***, Same as ***F*** but for average velocity.

To assess transfection of the virus and placement of the cannula, sections were visualized using a ﬂuorescent Axioskop 2 Plus microscope (Zeiss) ﬁtted with a CoolLED pE-100 excitation system and images acquired using the AxioVision software. Transfection of the virus in the LCN was assessed as follows: cerebellar sections were mapped onto standardized sagittal sections of a rat brain using a stereotaxic atlas (Paxinos and Watson, 2007). Sections at key anatomical points 0.18, 0.9, 1.4, 1.9, 2.4, 2.9, 3.4, 3.9, 4.2, and 4.6 mm from midline were identiﬁed and used for manual scoring of ﬂuorescence intensity. Fluorescence intensity was scaled from 0 to 5, with 0 representing no ﬂuorescence and 5 maximal ﬂuorescence across sections. In each animal, sections with maximum and minimum ﬂuorescence were determined by comparing the sections to each other while keeping illumination settings constant. The section with maximum ﬂuorescence was determined as the section with the largest ﬂuorescent halo around the injection site. Then each section was divided into three regions: cerebellar cortex, nuclei, and white matter. A ﬂuorescence score was determined per region by comparing within and across sections. To determine the spread of ﬂuorescence in the mediolateral direction, we used the ﬂuorescence scores of the nuclei to visualize the spread of the injection (Fig. 1).

From these data, several quantitative measures of viral expression were derived for subsequent analyses. Mean fluorescence intensity across all transfected cerebellar nuclei was calculated to provide an overall index of expression strength. A laterality index, reflecting hemispheric asymmetry, was computed as (right − left) / (right + left) using the mediolateral distance from the midline to classify hemispheres. The spatial extent of expression was defined as the difference between the maximum and minimum mediolateral positions where expression intensity exceeded zero (max distance − min distance). Finally, an asymmetry index capturing lateral bias normalized by overall spread was calculated as (max right − max left) / total spread. These quantitative descriptors were then used to examine potential relationships between viral expression patterns and behavioral performance.

### Behavioral measures

#### Open ﬁeld

A DeepLabCut model (Mathis et al., 2018) was trained to track animals’ movement in the open ﬁeld using the position of the head. A custom-written MATLAB script (version 2021a) was used to extract the total distance traveled as well as the average velocity.

#### Interval timing task

In order to test if chemogenetic manipulation of cerebellar circuits affected the rats’ ability to perform the interval timing task, three metrics were calculated: (1) overall task performance (Eq. 1), the percentage of correct trials, calculated as the number of trials in which the cue was presented and the rat exited the hold port in the reward window, over the total number of trials that the rat initiated, calculated as follows:

$$\;\mathrm{Performance}(\text{\%})=\frac{N_{2.25<t_{\mathrm{exit}}<3.5}}{N_{\mathrm{total}}}\times 100,$$

(2) exit time for each trial (Eq. 2), time of hold port release released subtracted by sound onset, both measures with respect to trial start, calculated as follows:

$$t_{\mathrm{exit}}(s)=t_{\mathrm{release}}-t_{\mathrm{onset}},$$

with *t*_(0)_ = trial start, and (3) reward latency for each correct trial (Eq. 3), measured as the time between exit from the hold port and nose poke in the reward port on correct trials, calculated, with *t*_(0)_ = trial start, as follows:

$$t_{\mathrm{reward}}(s)=t_{\mathrm{reward}}-t_{\mathrm{release}}.$$

All behavioral measures were extracted from the raw Klimbic ﬁle from each session via custom-written scripts in MATLAB (version 2021a) and Python (version 3.9).

### Statistical analysis

To assess the effect of CNO on the behavioral metrics of the interval timing task, generalized linear models (GLMs) and linear mixed models (LMMs) were employed. These statistical techniques take into consideration multiple levels of correlation in the dataset (e.g., when collecting multiple trials over different sessions). LMMs were used in the case of normally distributed data, whereas generalized LMMs (GLMMs) were used for data following non-normal distributions. LMMs were fit using the lmer function from the lme4 package in R (version 2024.04.2-764). The model included fixed effects for group, manipulation, and their interaction and random effects for session date, trials, and rat to account for repeated measures within sessions and individual differences among rats. The significance of fixed effects was evaluated using analysis of variance (ANOVA) with Satterthwaite's method. GLMs were fit with a binomial distribution and logit link function. We also used LMMs to build a null model that compared the cued trials from the predictable stage of the experiment with the cued trials from the unpredictable stage of the experiment under CNO conditions. Estimation plots which display the mean difference between groups with 95% bootstrap confidence intervals (CI) were used to show effect sizes (Ho et al., 2019) using the DABSET package in Python (version 3.9). A permutation test was also performed using the DABEST package with default parameters, which computes an empirical null distribution by randomly shuffling group labels and comparing the observed effect size to this distribution to derive a permutation *p* value. For open-ﬁeld analysis, an unpaired *t* test was used to compare the control and hM4D(Gi) virus group following a systemic injection of CNO. Data are presented as mean ± SD or 95% CI.

### Code and data availability

All data and code used in this study are freely available at https://github.com/BovenE/Bidirectional-Cerebellar-Control-of-Supra-Second-Timing-in-Rats/tree/master.

## Results

### Targeting of lateral cerebellar output

Human imaging and patient studies have indicated that regions of the cerebellar hemisphere are implicated in interval timing (Gooch et al., 2010; King et al., 2019). Given that the LCNs are the main output of the cerebellar hemispheres in rats we targeted infusion of inhibitory DREADD virus, AAV5-hSyn-hM4D(Gi) [termed hM4D(Gi)] or control virus AAV5-hSyn-EGFP (which lacks the DREADD receptor), into LCN bilaterally, to chronically manipulate cerebellar output during an interval timing task (Fig. 1*A*). Successful targeting was supported by the presence of hM4D(Gi)-mCherry or control EGFP expressed in the LCN (Fig. 1*B*). While there was some expression in the cerebellar cortex, this was variable across animals and was not related to any interanimal differences in behavior. Semiquantitative mapping of the expression across the cerebellar nuclei (Fig. 1*C*,*D*) showed that expression was generally centered on but not restricted to the LCN.

### Chemogenetic manipulation of cerebellar output affects general motor performance

As a first step, it was important to assess whether chemogenetic inhibition of LCN had an effect on general motor performance. To this end, we compared the effect of systemic CNO administration on open-field exploration on the hM4D(Gi) group (*n* = 6 rats) compared with the control EGFP group (*n* = 5 rats; Fig. 1*E*). Open-field performance was assessed 30 min after the administration of CNO. The hM4D(Gi) group, compared with control, showed a significant reduction in open-field exploration, expressed as the total distance traveled [Fig. 1*E–G*; hM4D(Gi) 29.45 ± 19.5 m; *n* = 6 rats; EGFP 61.37 ± 11.71 m; mean ± SD; *n* = 5 rats, *p* = 0.0063, unpaired *t* test], as well as a reduction in the average velocity during movement [Fig. 1*G*; hM4D(Gi) = 5.63 ± 3.75 cm/s; *n* = 6; EGFP = 12.78 ± 2.0 cm/s; *n* = 5; *p* = 0.0021]. These results provide a positive control that CNO activation of DREADD-transfected neurons in the cerebellar nuclei was effective but that general motor effects may be a confound in the interval timing task.

### Interval timing task

**Figure 2.**
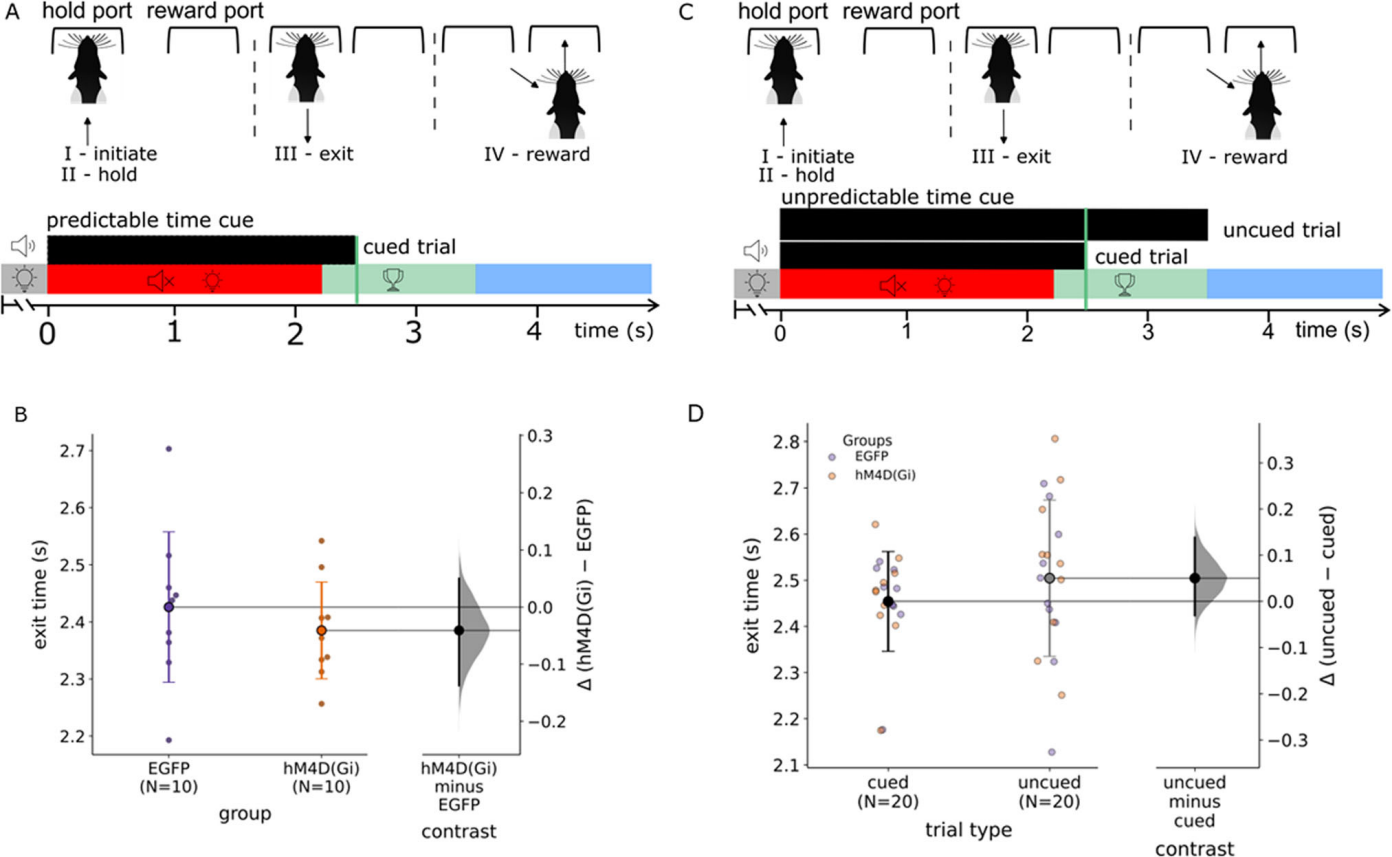
Rats perform an interval timing task. ***A***, Task structure indicating the action sequence (I–IV; top) during a trial in the interval timing task with predictable time cue. The timeline (bottom) indicates the trial sequence of possible stimulus events and trial outcomes in the operant chamber. A rat initiates a trial by poking into the hold port (I), triggering, after a random delay, an auditory stimulus of 2.5 s (black bar), which remains the same across the session and therefore the cue becomes predictable, during which the rat fixates its nose into the hold port (II). The trial outcome depends on when the rat exits the hold port, which is measured by the exit time (III). The gray bar indicates the time period in which an exit is too early; the red bar indicates the time period in which an incorrect trial occurs if the rat exits after stimulus onset but before the start of the reward window and results in the sound turned off and stimulus lights on, and the green bar indicates a correct trial in which a reward can be triggered in the reward port (IV). The time it takes for the rat to move from the hold port to the reward port is further referred to as the reward latency. The blue bar indicates the time period of too late trials in which the rat did not exit from the hold port in the reward window. The rat will receive a reward only if it exits the hold port within the reward window (light green). ***B***, Estimation plots [mean difference in exit times between groups (Δ), 95% bootstrap CI], comparing exit times between control (EGFP, *N* = 10) and experimental [hM4D(Gi), *N* = 10] groups during the predictable cue task. The permutation test found no statistical difference between the two experimental groups (*p* = 0.44). ***C***, Interval timing task with randomly interleaved cued and uncued trials, in which the action sequence remains the same as in ***A***; however, an unpredictable time cue (top black bar) which is represented by the longer black bar is also presented in 50% of the trials. ***D***, Left panel, Exit time across all animals (*n* = 20) during an interval timing session with randomly interleaved cued (2.5 s) and uncued (3.5 s) trials. Estimation plot [mean difference in exit time (Δ), 95% bootstrap CI], showing mean holding times for cued (*N* = 20) and uncued (*N* = 20) trials across both EGFP (purple) and hM4D(Gi) (orange) groups. The permutation test found no statistically significant difference between the trial types (*p* = 0.267).

To investigate how rats estimate and utilize both externally and internally cued temporal intervals to guide behavior, we used an interval timing task with predictable and unpredictable time cues (Fig. 2). Specifically, the study compared when the timing of a reward could be anticipated based on an external cue (Fig. 2*A*, predictable time cue) versus when the external cue duration was variable and the reward timing could not be directly inferred from cue offset (Fig. 2*C*, unpredictable time cue). The predictable condition assessed the animals’ ability to associate a fixed 2.5 s auditory cue with a reward window, testing temporal precision in response to a consistent auditory signal (Fig. 2*A*). Conversely, the unpredictable condition evaluated the rats’ capacity for self-timing independent of external sensory cues by incorporating trials with a 3.5 s tone and the same reward window as for the predictable time cue condition (Fig. 2*C*). This design enabled the distinction between externally driven stimulus-based timing and internally generated temporal estimation mechanisms. Top–down webcam monitoring and IR beam detection confirmed that during the hold interval, rats maintained a stable head position within the port with minimal whole-body movement. Although we did not quantify micromovements (e.g., whisking), the requirement to hold the head in place minimized the use of stereotyped or whole-body timing behaviors that can accompany timing when movement in rats is unconstrained (Fetterman et al., 1998; Gouvêa et al., 2014).

Rats were first trained on the predictable time cue task (cued trials). The cue consisted of a white noise stimulus. Each trial was initiated by the rat poking its nose into the hold port. After a random delay of 0.5–1.5 s, a sound with a fixed duration of 2.5 s was presented which served as a predictable cue for the rat to exit from the hold port at sound offset (Fig. 2*A*). The rat was rewarded with a food pellet if it exited the hold port during the reward window 2.25–3.5 s from the start of the sound cue. The rats could learn this interval timing behavior with predictable time cue after ∼7 d of training. When trained (>50% accuracy on two consecutive sessions and <30% too early responses), individual animals displayed exit times that were tightly clustered around the reward time interval. Across all animals, exit times were centered near 2.4 s, with group means of 2.43 s for EGFP and 2.39 s for hM4D(Gi) animals (Fig. 2*B*). There was no statistically significant difference in the exit times between the two experimental groups as determined by a permutation test (*p* = 0.44).

After rats were trained and tested on the interval timing task with the predictable time cue (cued trials), they moved on to sessions where cued trials occurred randomly in 50% of the trials. In the other 50% of trials, the same sound was played but for a longer duration (3.5 s; Fig. 2*C*). The latter are referred to as uncued trials because exit from the hold port was not cued by offset of the sound. Uncued trials had the same reward window as that used in cued trials. Therefore, in these sessions, uncued trials tested if rats (based on prior experience and reinforcement), could reliably estimate the time interval from the sound onset to exit the hold port to receive the reward.

After animals were trained on the interval task with both cued and uncued trials (>50% accuracy on two consecutive sessions and <30% too early responses), the mean group exit time for cued trials showed a distribution similar to that observed in the interval timing task with predictable cue trials alone (Fig. 2*D*), with a mean exit time of 2.45 s and a similar mean exit time for the uncued trials of 2.50 s. For individual animals, the distribution of exit times in response to the cued and uncued trials showed a similar pattern of exit times centered ∼2.5 s (Fig. 2*D*). There was no statistically significant difference in the exit times between the two trial types (Fig. 2*D*; permutation test, *p* = 0.267). This indicates that the animals could perform the interval timing task for cued and uncued trials with similar success.

### Chemogenetic manipulation during predictable timing delays action initiation

**Figure 3.**
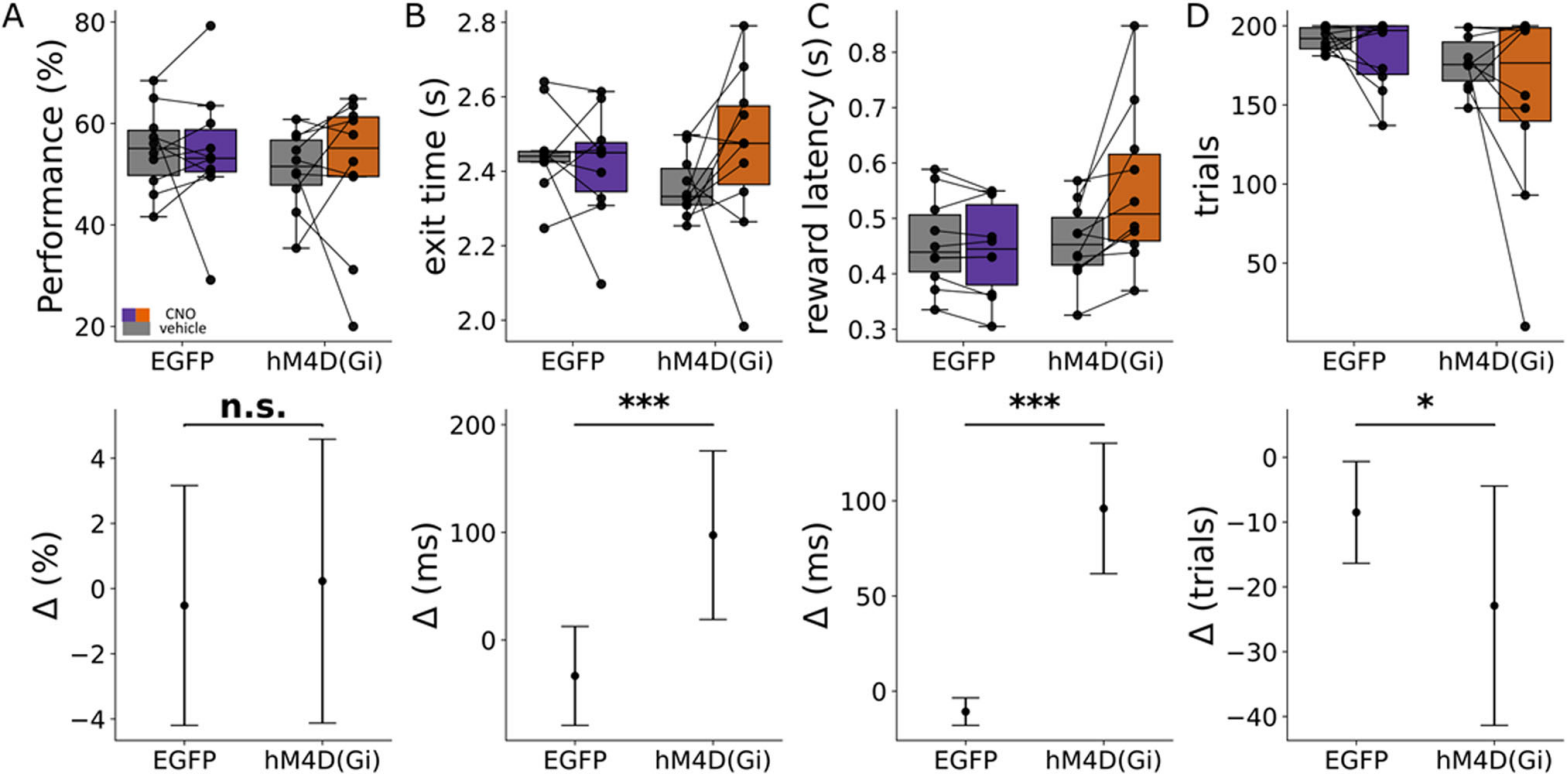
Effect of cerebellar manipulation during the interval timing task with predictable time cue. ***A***, Boxplots of the performance showing the group summary statistics [the box shows the middle 50% of the data (interquartile range), with the line inside representing the median. Whiskers extend to the smallest and largest values within 1.5 times the interquartile range] and the distribution of individual mean exit times during one session of the interval timing task with predictable time cue when given CNO (orange, *n* = 10) or vehicle (gray, *n* = 10) in each virus group. The lower plot shows the effect size shown as the average change in performance between vehicle and CNO for EGFP and hM4D(Gi) groups. ***B***, Boxplots and effect size for exit time, plotted in the same way as in ***A***. ***C***, Boxplot and effect size for the reward latency. ***D***, Boxplots and effect size for the number of trials. *p < 0.01; **p < 0.001; ***p < 0.0001.

When rats achieved stable performance in the predictable time cue task, CNO or vehicle was injected intraperitoneally on separate sessions with one session per condition (see Materials and Methods). First, we assessed if CNO treatment affected task performance (defined as the proportion of correct trials where the rat exited the hold port within the reward window expressed as a percentage of the total number of initiated trials within one session). A GLMM was fitted to the data, where reward outcome was the dependent variable and manipulation (vehicle or CNO) and group [EGFP or hM4D(Gi)] were the independent variables. No statistically significant difference was found for the interaction between group and manipulation, indicating that there was no observable effect of CNO on task performance (95% CI [−0.120, 0.254]; *p* = 0.485; Fig. 3*A*). Next, to assess if CNO treatment affected the time taken for the rat to exit from the hold port, an LMM model was fitted to the data, with exit time as the dependent variable, manipulation and group as the independent variables, and animal ID, trials, and session date as the random terms. There was a statistically significant effect of the interaction between group and manipulation, with an average increase of 150 ± 27 ms in exit time for the hM4D(Gi) group following injection of CNO (95% CI [97.45, 204] ms; *F* = 30.703; *p* < 0.001; Fig. 3*B*). Similarly, there was a statistically significant effect of the interaction between CNO and group on reward latency (Fig. 3*C*), with an average increase of 80 ± 6 ms (95% CI [67.57, 90] ms; *F* = 189.468; *p* < 0.0001) for the hM4D(Gi) group following CNO injection. The total number of trials executed in the operant box was analyzed using a GLM with the number of trials per session as the dependent variable and the group and manipulation as the independent variables to determine if CNO manipulation affects overall ability to perform the task (Fig. 3*D*). There was also a statistically significant reduction in the total number of trials performed by hM4D(Gi) rats following CNO administration, with animals completing ∼10% fewer trials compared with the EGFP group (95% CI [−0.1961, −0.0093]; *F* = 4.64; *p* = 0.031). In summary, these results indicate that although cerebellar manipulation has no detectable effect on overall success rate in performance of the task, it induces slowing of nose-poke movement, resulting in delayed exit time and increased reward latency as well as a moderate reduction in the number of trials performed.

### Chemogenetic manipulation during unpredictable timing advances action initiation

The same animals were subsequently exposed to one CNO and one vehicle session (see Materials and Methods) in which the tone duration between trials was varied randomly, either with a tone duration of 3.5 s (uncued trials) or 2.5 s (cued trials) with the reward time window in both cases occurring between 2.25 and 3.5 s (as in the predictable time cue task). In each session, the number of cued and uncued trials were balanced and randomized.

**Figure 4.**
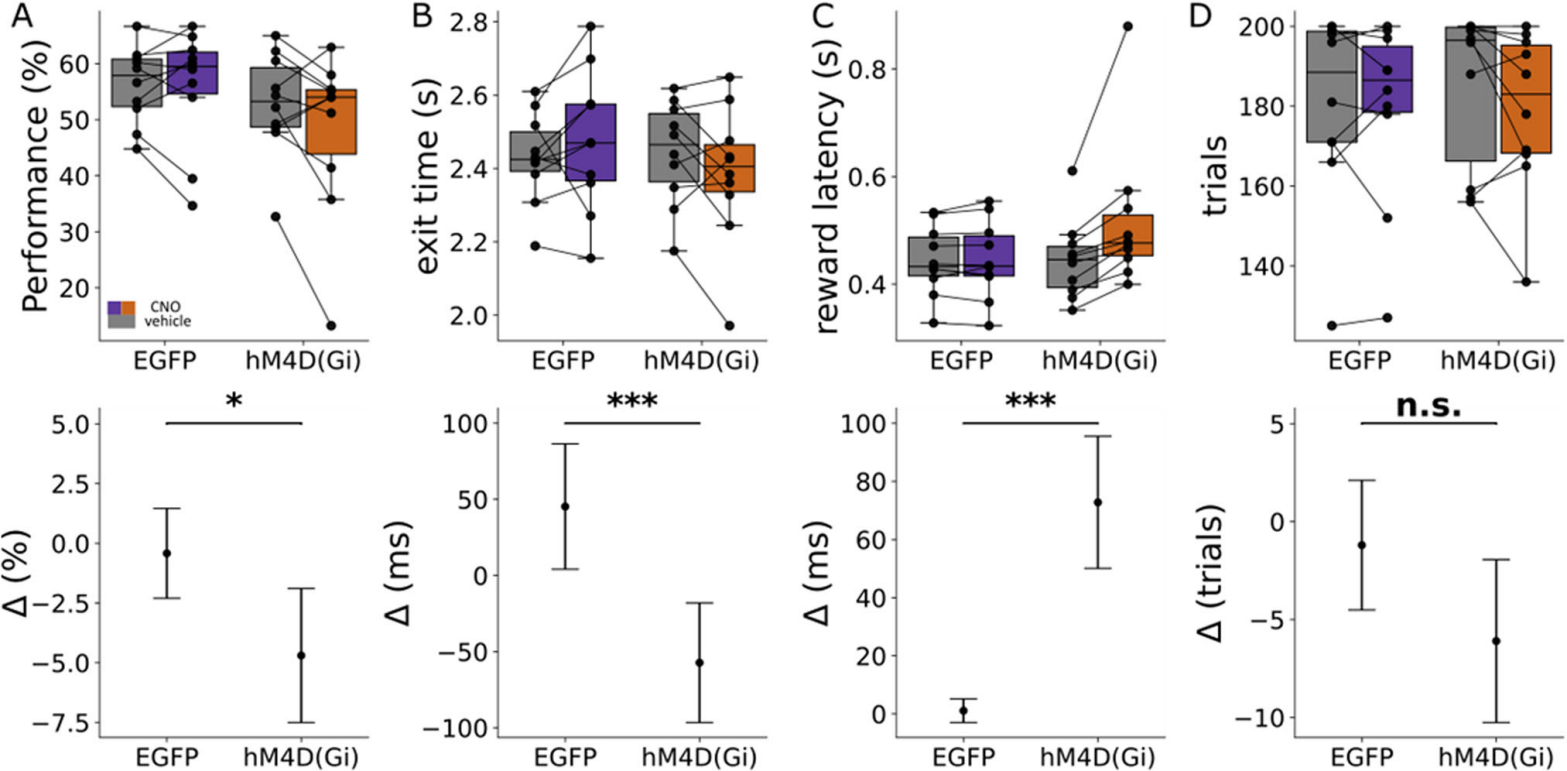
Effect of cerebellar manipulation during the interval timing task with unpredictable time cue. ***A***, Boxplots of the performance showing the group summary statistics and the distribution of individual mean exit times during one session of the interval timing task with unpredictable time cue when given CNO (orange, *n* = 10) or vehicle (gray, *n* = 10) in each virus group. ***B***, Boxplots of the exit time plotted in the same way as in ***A***. ***C***, Boxplots and effect size for the reward latency. ***D***, Boxplots and effect size for the number of trials. *p < 0.01; **p < 0.001; ***p < 0.0001.

To assess if cerebellar manipulation affected task performance, when considering all trial types, we fitted a GLMM to the data, with reward outcome as the dependent variable and manipulation and group as fixed factors. There was a statistically significant effect of the interaction between group and manipulation, with the hM4D(Gi) group performing on average 4.8% worse than control when given CNO (95% CI [−0.379, −0.010]; *z*  = −2.069; *p* = 0.039; Fig. 4*A*). To assess if cerebellar manipulation affected the time it takes for the rat to exit from the hold port, an LMM was fitted to the data, with exit time as the dependent variable and manipulation and group as the independent variables, with rat ID, trial number, and session date as the random term. There was a statistically significant effect of the interaction between group and manipulation, with an average decrease of 107.95 ± 29.33 ms [95% CI [166.60, 49.329]; *F* = 13.544; *p* = 0.0002) in exit time for the hM4D(Gi) group with CNO (Fig. 4*B*). Despite exiting sooner, these premature responses did not translate into faster task completion; on the contrary, there was a statistically significant effect of the interaction between manipulation and group on reward latency, with an average increase of 67.6 ± 0.61 ms (95% CI [55.62 79.58]; *F*  = 122.612; *p* < 0.0001) for the hM4D(Gi) group upon administration of CNO (Fig. 4*C*). The total number of trials executed in the operant box was analyzed using GLMM with the total number of trials per session as the dependent variable and group and manipulation as the independent variables and rat ID as the random effect. Results indicated that there was no statistically significant difference in the total number of trials performed (95% CI [−0.102, 0.081]; *p* = 0.819; Fig. 4*D*). Together, these results suggest that cerebellar manipulation during the unpredictable time cue task disrupts internal timing processes and reduces overall success rate in performance, primarily because rats exit the hold port prematurely yet delayed their reward retrieval.

To examine whether the CNO-related changes observed in the unpredictable stage of the experiment could reflect normal variability rather than a true effect of the CNO, a null LMM model was constructed to quantify the expected range of exit time variation under conditions of no manipulation effect. Cued trials from the first stage (predictable sessions) were compared with cued trials from the second stage (sessions containing both cued and uncued trials). Exit time was specified as the dependent variable, while manipulation (vehicle or CNO), stage (predictable or unpredictable), and viral group (EGFP or hM4D(Gi)) were the fixed effects, with rat and session date included as random effects. No statistically significant difference between manipulation and stage was found (*β* = 0.051, i.e., 51 ms; 95% CI [−0.009, 0.111]; *p* = 0.096), indicating that CNO did not differentially affect exit times between cued trials in the predictable and unpredictable stages of the experiment. The estimated mean difference of ∼50 ms was within the natural variability or “noise” captured by this null model. In contrast, the effect size observed in the main experimental condition was about double this variability (∼108 ms), supporting the proposition that the timing shifts observed with CNO are due to the experimental treatment.

Comparable GLMMs were fitted for the number of trials (*β* = −0.034; 95% CI [−0.155, 0.088]; *p* = 0.065), task performance (*β* = 0.014; 95% CI [−0.244, 0.273]; *p* = 0.065), and reward latency (*β* = 1.04; 95% CI [−0.45, 2.533]; *p* = 0.623), and no statistically significant interactions were found between experimental manipulation (CNO and vehicle) and trial stage (predictable vs unpredictable).

Taken together, these analyses therefore estimate the baseline variability in timing of task performance. Since the CNO-mediated effects reported in Figures 3 and 4 exceed this baseline by more than twofold, it seems reasonable to suggest that the effects observed were due to the experimental manipulation rather than natural variation in performance.

With regard to other possible reasons for variability in behavioral performance, one possibility is interanimal differences in viral expression at the cerebellar injection site. Cross-correlations were therefore performed between average hM4D(Gi) expression intensity, laterality index, total spread and asymmetry index, and key behavioral measures (performance, number of trials, exit time, and reward latency). Pearson's correlation revealed no systematic relationships between any of these anatomical and behavioral measures (all comparisons *p* > 0.05, data not shown). The exception was a negative correlation between change in trial number and average intensity (*r* = −0.65; *p* = 0.042). Overall, these results therefore suggest that interanimal variability in injection-site expression is not a major determinant of the observed variation in behavioral performance.

**Figure 5.**
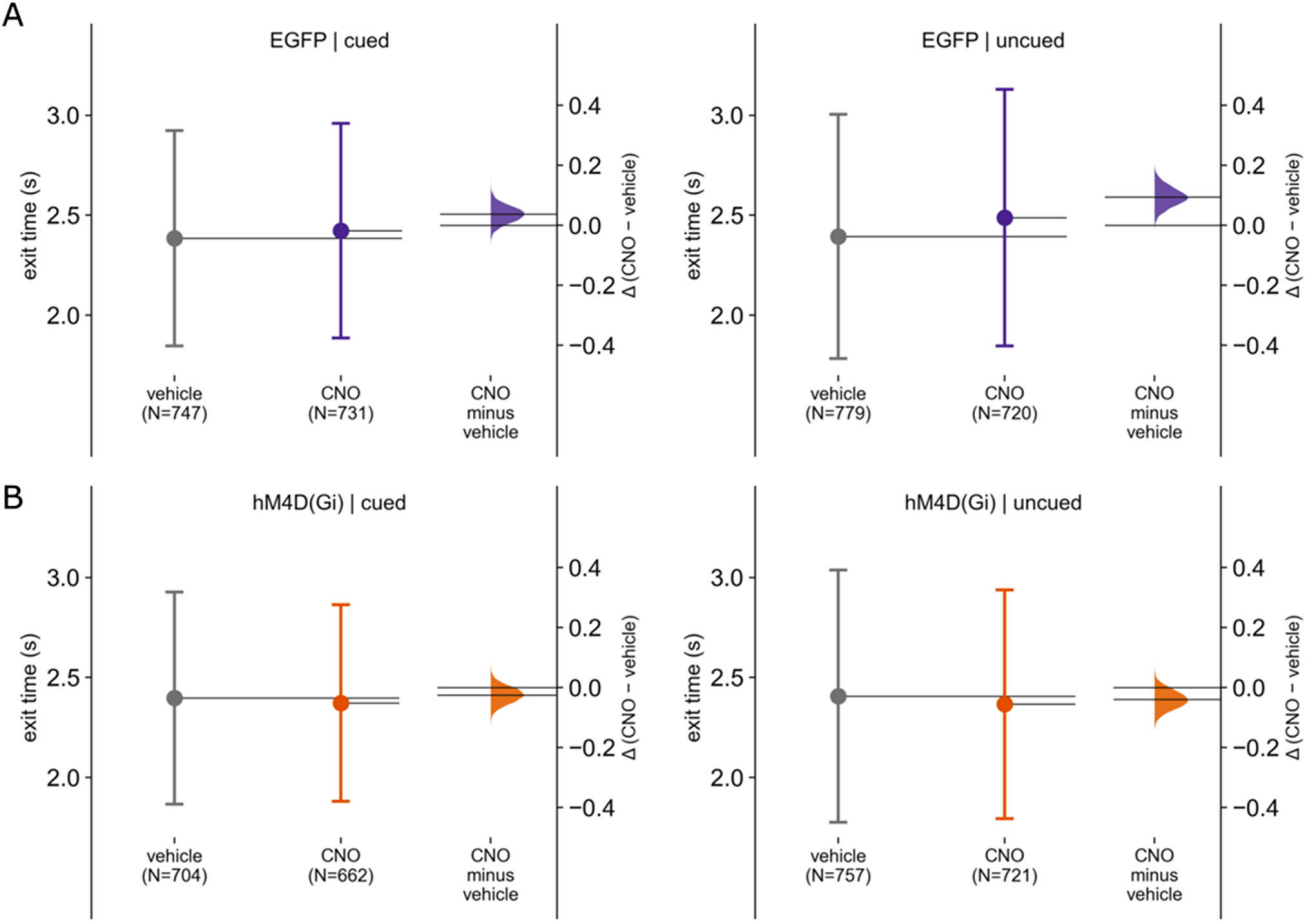
Effect of cerebellar manipulation on exit times during cued and uncued trials. Estimation plots [mean difference in exit time (Δ), 95% bootstrap CI], showing exit times in ***A*** EGFP and ***B*** hM4D(Gi) animals under vehicle (gray) and CNO [purple for EGFP, orange for hM4D(Gi)] conditions. Left panels display cued trials (2.5 s tone), and right panels show uncued trials (3.5 s tone).

Finally, the experimental design of the unpredictable time cue task allowed assessment of whether there was any difference in performance in cued and uncued trials obtained in the same session. First, to assess if cerebellar manipulation affects the time it takes for rats to exit from the hold port, we fitted an LMM to the data for the cued trials only, with exit time as the dependent variable and manipulation and group as the independent variables, with rat ID, trial number, and session date as the random term. There was no statistically significant effect of the interaction between manipulation and group on exit time (95% CI [−124.8, 0.002]; *p* = 0.059; Fig. 5*A*,*B*). In contrast, when only uncued trials were considered from the same recording sessions, there was a statistically significant effect of the interaction between group and manipulation, with an average decrease of 156.44 ± 40 ms (95% CI [−81, −232.15]; *F*  = 17.082; *p* < 0.001) in exit time for the hM4D(Gi) group with CNO compared with EGFP with CNO and controlled for manipulation. These results therefore suggest that with the experimental conditions used in the present study, cerebellar manipulation affects internally cued but not externally cued behavior in a suprasecond interval timing task in rats.

## Discussion

The current study provides evidence that inhibiting output from the cerebellum, specifically via the LCN, results in behavioral deficits in interval timing in rats. These deficits differed depending on the predictability of the time cue: cerebellar manipulation caused delayed exit from the waiting port when the time cue was predictable, whereas when the time cue was unpredictable, it led to the opposite effect—premature exit times. It seems reasonable to conclude that this reduction in exit time was not primarily due to general motor deficits and alternative behavioral strategies for the following reasons: (1) a cardinal sign of cerebellar dysfunction is a slowing of voluntary movements, including increased reaction times (Ito, 1984). In the present study, the cerebellar manipulation resulted in slowing of locomotion in the open field and slowing of nose-poke movement in the reward phase of the predictable and unpredictable cued tasks. The premature exit times in the time estimation phase of the task were therefore specific to unpredictable time cue sessions, indicating the effect was not a generalized motor deficit. (2) The shifts in response timing occurred without loss of overall task success, suggesting selective disruption of temporal computation rather than generalized motor impairment. (3) Rats display stereotypical behaviors such as self-grooming that may be used to pace time (Fetterman et al., 1998; Gouvêa et al., 2014). While movements such as licking and chewing cannot be excluded as a timing strategy, the use of a nose-poke hold task meant that the animals’ behavioral repertoire was constrained and movements such body grooming and locomotion were not available to the animals to pace time. It therefore seems reasonable to suggest that the premature exit times were mainly, if not exclusively, due to an impairment in internal representation of timing. However, future work combining hold-based timing with high-resolution whole-body movement tracking will be valuable for determining if more subtle behaviors can be excluded.

### Magnitude and biological relevance of timing effects

While the observed effects on timing behavior were on average a small fraction of 1 s (100–160 ms), multiple lines of evidence suggest their potential biological relevance. First, the null model demonstrated that natural variability in exit times under CNO was ∼50 ms; the experimentally induced timing shifts observed in our study exceed this baseline by more than twofold, supporting the conclusion that they reflect true experimental effects rather than random fluctuations in behavioral performance. Second, the magnitude of effect is consistent with cerebellar timing across species: primate studies show that dentate nucleus microstimulation advances self-timed saccades by ∼100 ms (Ohmae et al., 2017), while muscimol inactivation delays movements by 100–200 ms and increases trial-by-trial variability (Kunimatsu et al., 2018). Third, as outlined above, the directionality of our effects—delayed exits in predictable trials and premature exits in unpredictable trials—further suggests a systematic shift in temporal estimation rather than random variance. The functional significance of altering timing precision within a 100–160 ms timeframe remains to be determined, but such effects could be important for rapid survival-relevant behaviors including predator evasion, prey capture, and coordinated social interactions (Buhusi and Meck, 2005; Coull et al., 2011).

The magnitude of our effects may, in part, reflect limitations of the chemogenetic approach, which allows for sustained modulation of neuronal activity rather than precise, real-time inhibition. Chemogenetic inhibition of the LCN engages heterogeneous local circuitry. In slice preparations, CNO reduces firing of hM4Di-expressing neurons; however, in vivo effects are more complex due to the presence of both excitatory projection neurons and inhibitory interneurons within the nucleus (Uusisaari et al., 2007). Consequently, broad chemogenetic inhibition can produce a variety of effects: silencing inhibitory interneurons may disinhibit their downstream targets, while silencing excitatory neurons directly suppresses their targets resulting in heterogeneous population-level responses (Locke et al., 2018). These network-level effects may contribute to both the graded behavioral changes and interanimal variability observed in our study. Although we examined several quantitative measures of viral expression including intensity, mediolateral spread, and asymmetry, we did not find systematic relationships between these metrics and individual behavioral effects. This suggests that variability may arise less from differences in expression patterns and more from differences in how chemogenetic inhibition interacts with the local microcircuit architecture of each animal's LCN. The source of this variability therefore remains unclear but likely reflects factors beyond transfection patterns, such as individual differences in baseline timing strategies and in the connectivity patterns between cerebellar and cortical timing networks.

### Comparison with previous studies

The findings from this study contribute to the ongoing debate regarding the cerebellum's role in self-timing across subsecond and suprasecond intervals. In monkeys, Ohmae et al. (2017) and Kunimatsu et al. (2018) showed that neurons in cerebellar dentate exhibit ramping activity when animals self-initiate saccades after both sub- and suprasecond delays, indicating that cerebellar output participates in timing across different temporal scales, with ramping activity initiated earlier and extending across the delay for subsecond intervals, whereas for longer, suprasecond intervals, neural ramping occurred later and aligned more closely with movement initiation. Perturbing dentate output in these studies altered both the timing and trial-by-trial consistency of the self-initiated movements, with greater variability present at suprasecond intervals, indicating that cerebellar activity contributes to the precise timing of an action (Ohmae et al., 2017; Kunimatsu et al., 2018). Our results in rats parallel the findings in primates by showing that chemogenetic inhibition of lateral cerebellar output disrupts both the timing and reliability of internally generated actions over seconds. Together, these findings support a view in which the cerebellum contributes to both subsecond motor timing and suprasecond cognitive timing, providing a scalable internal timing signal that governs the initiation and precision of self-timed actions across species and tasks, rather than acting solely as a downstream executor of cortical timing commands (Ohmae et al., 2017; Kunimatsu et al., 2018; Tanaka et al., 2024).

The findings in rats of Heskje et al. (2020) are consistent with the present work. Pharmacological inhibition of D1 dopamine receptors in the LCN led to increased variability and imprecise timing in a suprasecond interval timing task where rats had to estimate elapsed time. Specifically, they observed a decrease in temporal accuracy, with animals responding earlier than required, mirroring the premature responses observed in our unpredictable timing condition following chemogenetic inhibition of the LCN. Furthermore, Heskje et al. (2020) performed electrophysiological recordings in the medial frontal cortex and showed that cerebellar D1 receptor inhibition disrupted ramping activity, a neural signature closely linked to interval timing performance (Buhusi and Meck, 2005; Narayanan, 2016). While the current study did not include electrophysiological recordings, the behavioral similarities observed suggest that cerebellar inhibition may impair self-timed responses through disruption of neocortical timing mechanisms.

Heslin et al. (2022) investigated in rats the role of the LCN in interval timing but in contrast to our findings did not find any significant effects. However, an important difference with the present study is that Heslin et al. (2022) considered longer time windows, ranging from 4 to 12 s. Another important difference was the type of behavior studied. While in the present study animals were required to keep their noses within the waiting port and then perform a discrete head movement to obtain a reward, Heslin et al. (2022) used a timing task where the animals were not restricted from carrying out movements such as self-grooming or pacing. As outlined above, such stereotypical behavior can be used to improve temporal judgments (Fetterman et al., 1998; Gouvêa et al., 2014). Nonetheless, the discrepancy with our findings merits further study in which factors such as the time interval employed (seconds vs tens of seconds) and type of movement are taken into account.

The findings from the current study are, however, broadly consistent with results obtained in cerebellar patients (Gooch et al., 2010). We found that timing impairments were bidirectional based on task demands. Similarly, patients with lateral cerebellar damage show increased errors in time production but decreased errors in time estimation. In the present study, one important difference between the predictable and unpredictable time cue tasks is that in the former there is no requirement for the animal to keep track of time, as the stimulus (auditory cue offset) and reward-related response are closely coupled. However, in the unpredictable time cue task, stimulus and reward are decoupled (Freestone and Church, 2010; Freestone et al., 2013). In terms of reward prediction, the former task can therefore be considered mainly an externally cued response whereas the latter is more an internally cued response.

### Internal models

A key question is how the cerebellum contributes to timing behavior across different timescales. Traditionally, the cerebellum has been framed within the internal model hypothesis, where it functions as a predictive system that anticipates the sensory consequences of motor actions and fine-tunes movements accordingly (Wolpert et al., 1995, 1998; Sokolov et al., 2017; Streng et al., 2022). While internal models have been extensively studied in subsecond motor control, recent evidence suggests that the cerebellum extends its predictive functions to longer timescales, integrating motor and sensory information to form internal representations of elapsed time. At the cellular level, cerebellar Purkinje cells have been shown to acquire internal models of suprasecond stimulus timing, utilizing prediction errors to refine these representations (Narayanan et al., 2024). In zebrafish, cerebellar circuits modulate decision timing and action selection, with cerebellar neurodynamics predicting movement onset >10 s in advance (Lin et al., 2020). At the network level, a computational model of cerebrocerebellar networks proposes that the cerebellum provides temporally extended feedback predictions to cortical recurrent networks, thereby facilitating learning and performance in tasks that unfold over hundreds of milliseconds to seconds (Boven et al., 2023). Together, these findings suggest that cerebellar internal models can contribute to long-term motor planning and decision-making.

In conclusion, the present study provides evidence in rats that the cerebellum's role in interval timing extends beyond control of movements within subsecond timescales to encompass temporal processing at longer time intervals associated with more complex tasks such as action planning that conventionally are considered to be regulated by higher-order structures. By integrating temporal processing across motor and cognitive domains, the cerebellum may support adaptive behavior in all its forms—from subsecond control involved in conditioned reflexes to suprasecond control involved in complex self-timing tasks. Future studies should determine if common cellular processes are involved across this wide temporal range and explore cerebellar interactions with brain-wide neural networks involved in timing and prediction.

## Synthesis

Reviewing Editor: David Schoppik, New York University Grossman School of Medicine Decisions are customarily a result of the Reviewing Editor and the peer reviewers coming together and discussing their recommendations until a consensus is reached. When revisions are invited, a fact-based synthesis statement explaining their decision and outlining what is needed to prepare a revision will be listed below. The following reviewer(s) agreed to reveal their identity: NONE.

1. The size of the effects reported in the manuscript are not especially large given the variance, and it would be helpful to have a null model to compare them to so that the reader has a sense for what sort of effects might be noise and what might be real. Fortunately, the design of the experiment suggests a way forward. We recommend building an LMM to directly compare the size of the CNO-mediated effects on cued trials from the first stage to the cued trials in the second stage (where there were both cued and uncued trials). To our mind there should be no difference in performance/exit time/reward latency/trials. The CNO-mediated changes for each give a helpful range of what "noise" looks like and, if indeed the changes observe in the analyses that comprise Figures 4/5 are greater, it would strengthen the conclusions considerably.

2. We don't agree with the claim that (Discussion) "general motor deficits...would be expected to increase exit time." The claim is predicated on a model that the animals are waiting to move. Often, rodents engage in stereotyped behaviors while marking time and it's not clear how those interact with the lesions as they aren't measured here. It's worth dedicating space in the Discussion to the limits of the manuscript's claim that motor deficits would always manifest as increased exit time.

3. The choice to show cumulative distributions in Figures 1 and 6 make it impossible for us to visually evaluate changes to the means (Fig 1) and claims about differences in the text for Fig 6; in particular, there's no way to evaluate variance visually. We recommend complementing these graphs with summary statistics. Generally eNeuro recommends estimation statistics https://www.eneuro.org/content/6/4/ENEURO.0205-19.2019 .

Minor:

p9 "+/- 140 lux" what is the +/- doing here? Is there a mean value for luminance missing?

p14 Please reorder the figures to match the main text, which now calls them as 2/3/1. Please also consider combining the figures; they are relatively sparse on their own, although this is ultimately up to the authors.

4. We are concerned whether a ~5% effect noted in Figure 5B is enough to claim that the manipulation leads to "premature responses when self-timing was required," as claimed in the abstract. Specifically, we wonder about effect size and relevance. The variability between animals seen in Figure 5B suggests either other variables contributing to the effect (discussed as limitations of DREADD) or simply that the biological effect is close to the noise floor. All things being equal, the paper could have been very conservatively written to state that there was a small, but perhaps biologically irrelevant alteration in capacity for internal timing.

We would be interested in the authors exploring the source of their variability in this task a bit more. Presumably, if the authors are correct, the extent or nature of the DREADD infection in Figure 2 should correlate with the effects in Figure 5B. The distribution of effect sizes for those animals varies almost bimodally, with some exhibiting a consistent 10-15% decrease in time while the other population exhibits minimal increases in time. If the source of these differences could be parsed out, the authors' arguments would be dramatically strengthened, and we would be more inclined to agree with their conclusions. Does more lateral CbN infection lead to more effects (could these be directly correlated)? Does more specific infection?

Minor: The methods state 10 animals were used for eGFP and DREADD groups, but I see 8 in Figure 2. The nature of those missing animals should be stated somewhere. Some discussion or citation from another study validating the use of DREADD in the DCN, and the expected changes in activity is warranted.

## Author Response

Point by Point Reply to Reviewers Comments 1. The size of the effects reported in the manuscript are not especially large given the variance, and it would be helpful to have a null model to compare them to so that the reader has a sense for what sort of effects might be noise and what might be real. Fortunately, the design of the experiment suggests a way forward. We recommend building an LMM to directly compare the size of the CNO-mediated effects on cued trials from the first stage to the cued trials in the second stage (where there were both cued and uncued trials). To our mind there should be no difference in performance/exit time/reward latency/trials. The CNO-mediated changes for each give a helpful range of what "noise" looks like and, if indeed the changes observe in the analyses that comprise Figures 4/5 are greater, it would strengthen the conclusions considerably.

We thank the reviewers for this suggestion and have now fitted a linear mixed model (LMM) comparing predictable stage cued trials to unpredictable stage cued trials under CNO manipulation. Since no systematic difference is expected between these trial types, any observed effect would reflect fluctuations likely attributable to chance to chance in our experimental design. In order to build a null model, we constructed the LMM, with exit time as the dependent variable and manipulation (vehicle or CNO), stage (predictable or unpredictable) and group (eGFP or hM4D(Gi)) as the independent variables with random effects of rat and session date. No significant difference was found for the interaction between manipulation and stage, indicating that there was no observable effect of CNO on exit time in cued trials across the stages (β= 0.051, 95% CI [-0.009, 0.111] p= 0.096). The estimated effect size of 51 ms and the estimated effect size in the experimental condition exceeds the noise floor defined by the control LMM model. We constructed a similar generalized mixed effect (GLMM) models for trials (β=-0.034, 95% CI [-0.155, 0.088] p= 0.065), performance (β=0.014, 95% CI [-0.244, 0.273] p= 0.065) and reward latency (β=1.04, 95% CI [-0.45, 2.533] p= 0.623) and did not find any statistical significant effects. Consistent with the reviewers' expectation, these additional analyses help strengthen our conclusion that the CNO-mediated effects are above noise levels. This additional analysis has been included in the manuscript (Page 13 line 306, Page 18, line 444).

Crucially, the CNO induced changes reported in what are now Figures 3 &4 are larger than this baseline noise level. For example, during the unpredictable stage average action initiation was delayed on the order of 100-160 ms under CNO, whereas the predictable stage vs unpredictable stage difference in the CNO effect (our null comparison) was near zero. In practical terms, the ~100 ms shift we observed with CNO exceeds what would be expected by chance alone, giving us confidence that this is an effect of the CNO and not an artifact. Indeed, the effects remain statistically significant in our analysis despite the underlying variance, indicating a consistent directional shift across animals. Thus, while the absolute magnitude of the effect is modest, it is larger than experimental noise.

2. We don't agree with the claim that (Discussion) "general motor deficits...would be expected to increase exit time." The claim is predicated on a model that the animals are waiting to move. Often, rodents engage in stereotyped behaviors while marking time and it's not clear how those interact with the lesions as they aren't measured here. It's worth dedicating space in the Discussion to the limits of the manuscript's claim that motor deficits would always manifest as increased exit time.

We have modified the text in the Discussion (p20) to make our conclusions more circumspect and acknowledge that stereotypical behaviours can be used by rodents to self-pace tasks. However, we also make the case that this possibility seems unlikely to be fully explain our findings because we used a port nose-poke hold which constrains movement during the timing interval. Rats were required to maintain the head position in the hold port (detected by an IR beam). Top-down webcam monitoring confirmed head fixation at the hold port, minimizing whole-body movements during the critical timing period. This has now been added to the Methods and Results (p 7, line 152 and p 15 line 355). Related fixation/hold paradigms use a similar strategy to limit locomotion and whisking; for example, in a continuous nose-poke vibrotactile task, trained rats kept their head static and did not whisk during the ~600 ms stimulus period (McDonald et al., 2014), demonstrating that nose-poke fixation can substantially suppress ongoing movement. We acknowledge that subtle movements (e.g., licking and chewing) may persist and were not quantified in our study (p 21 line 503).

3. The choice to show cumulative distributions in Figures 1 and 6 make it impossible for us to visually evaluate changes to the means (Fig 1) and claims about differences in the text for Fig 6; in particular, there's no way to evaluate variance visually. We recommend complementing these graphs with summary statistics. Generally eNeuro recommends estimation statistics https://eur01.safelinks.protection.outlook.com/.

We have now included the estimation statistics (mean differences with 95% CIs) and used these in place of the cumulative distribution curves. The results text has been edited to reflect this.

4. We are concerned whether a ~5% effect noted in 5B is enough to claim that the manipulation leads to "premature responses when self-timing was required" as claimed in the abstract. Specifically, we wonder about effect size and relevance. The variability between animals seen in 5B suggests either other variables contributing to the effect (discussed as limitations of DREADD) or simply that the biological effect is close to the noise floor. All things being equal, the paper could have been very conservatively written to state that there was a small, but perhaps biologically irrelevant alteration in capacity for internal timing.

We have revised the abstract to specify the magnitude of the effect on exit time on uncued trials (~100-160ms). See also point 1 above in relation to the null LMM and noise floor and point 5 in terms of viral variability. An additional section to reflect these points has been added to the discussion (p 21, line 511). We have also included an explanation that silencing the lateral nucleus can produce heterogeneous downstream effects (both inhibition and excitation, see Locke et al., 2018), which has implications for behavioral variability. We have also modified the paper to be more conservative in our conclusions and have explicitly addressed inter-animal variability (p 22, line 540).

5. We would be interested in the author's exploring the source of their variability in this task a bit more. Presumably, if the authors are correct, the extent or nature of the DREADD infection in Figure 2 should correlate with the effects in 5B. The distribution of effect sizes for those animals varies almost bimodally, with some exhibiting a consistent 10-15% decrease in time while the other population exhibits minimal increases in time. If the source of these differences could be parsed out, the author's arguments would be dramatically strengthened, and we would be more inclined to agree with their conclusions. Does more lateral CbN infection lead to more effects (could these be directly correlated)? Does more specific infection? As suggested by the reviewers, we have explored one potential source of the variability in the task by considering differences in viral infection (hMD4(Gi) in relation to the behavioural effects in 5B). We have correlated the following measures to performance, trials, exit time and reward latency:

1. Average Intensity: mean hM4D(Gi) virus expression intensity across all transfected cerebellar nuclei areas regions per animal.

2. Lateral Index: A laterality score reflecting asymmetry of expression, computed from region-wise intensities as: (Right - Left) / (Right + Left), using mm distance from midline to classify hemispheres.

3. Total Spread: The spatial extent of expression, defined as the difference between the maximum and minimum medio-lateral positions where expression intensity was greater than zero: max(distance) − min(distance) 4. Asymmetry Index: A derived measure of expression imbalance, defined as: (max_right − max_left) / total_spread, capturing lateral bias normalized by spread.

We were unable to find any systematic relationship between any of these injection site measures and the performance measures (Pearson's correlation, all p > 0.05, except for a single negative correlation between Δ trials and average intensity, r = −0.65, p = 0.042). Overall, these analyses indicate that inter-animal variability in behaviour could not be accounted for by quantifiable differences in injection site expression. We have included these additional findings in the Results (p 19, line 465) and added further comment in the Discussion that the basis for the inter-animal variability remains unclear (page 22, line 540).

Minor:

6. The methods state 10 animals were used for eGFP and DREADD groups, but I see 8 in figure 2. The nature of those missing animals should be stated somewhere.

This has now been corrected with the additional animals added to the figure (now figure 1) 7. Some discussion or citation from another study validating the use of DREADD in the DCN, and the expected change in activity is warranted.

See response to point 4.

8. p9 "+/- 140 lux" what is the +/- doing here? Is there a mean value for luminance missing? The reviewer is correct: 140 lux denotes the measured mean illuminance. The +/- symbol was used to indicate that this was the mean lux value; we have removed the symbol.

9. Please reorder the figures to match the main text, which now calls them as 2/3/1. Please also consider combining the figures; they are relatively sparse on their own, although this is ultimately up to the authors.

We have combined figures as suggested. We have reordered the figures as presented in the results.
